# Habitat heterogeneity affects predation of European pine sawfly cocoons

**DOI:** 10.1002/ece3.3632

**Published:** 2017-11-12

**Authors:** Davide Bellone, Maartje J. Klapwijk, Christer Björkman

**Affiliations:** ^1^ Department of Ecology Swedish University of Agricultural Sciences Uppsala Sweden

**Keywords:** biological control, forestry, habitat diversity, natural enemy, outbreaks, population dynamics

## Abstract

Habitat heterogeneity is thought to affect top‐down control of herbivorous insects and contribute to population stability by providing a more attractive microhabitat for natural enemies, potentially leading to reduced population fluctuations. Identifying the parameters that contribute to habitat heterogeneity promoting top‐down control of herbivorous insects by natural enemies could facilitate appropriate management decisions, resulting in a decreased risk of pest insect outbreaks because of a higher level of predation. In our study, we measured the top‐down pressure exerted by small mammals on the cocoons of a notorious pest insect in pine forests, the European pine sawfly (*Neodiprion sertifer*), which is known to be regulated by small mammal predation. The forest stands used differed in heterogeneity measured in terms of differences in tree diversity and density, understory vegetation height, presence/absence, and density of dead wood. We found higher predation in more dense spots within forest stands. Further, the effect of dead wood on sawfly cocoon predation depended on the pine proportion in forest stands. The addition of dead wood in a manipulation experiment had a slight positive effect on cocoon predation, while dead wood removal caused a clear decrease in predation rate, and the decrease was more pronounced when the proportion of pine increased. Our results show that habitat heterogeneity affects predation by generalist predators on herbivorous insects. This knowledge could be applied to reduce the risk of insect outbreaks by applying management methods that increase heterogeneity in perennial systems such as forests and orchards, thus decreasing the levels of insect damage.

## 1 INTRODUCTION

It is widely recognized that habitat heterogeneity plays an important role in the interactions between plants, herbivores, and their natural enemies (Price et al., 1980). In recent decades, diversification in agricultural fields and forest stands has been considered one way to increase the biological suppression of potential insect pests (Letourneau et al., 2011; Simon, Bouvier, Debras, & Sauphanor, 2010). More heterogeneous habitats are thought to be more resilient to disturbances caused by pest insects that exhibit strong fluctuations in their population dynamics (Thompson et al., 2014). It has been suggested that heterogeneous habitats provide conditions promoting mechanisms that could reduce pest population fluctuations (Oliver, Roy, Hill, Brereton, & Thomas, 2010) and may result in reduced plant damage.

Underlying mechanisms that could affect the dynamics of insect pests in relation to habitat heterogeneity are (1) bottom‐up processes related to host plant quality and (2) top‐down processes in terms of the rate of mortality imposed by natural enemies (Haddad et al., 2009). In this study, we focus on how pressure exerted by natural enemies might be affected by habitat heterogeneity. According to the enemy hypothesis, heterogeneous habitats may favor an increase in the abundance and diversity of natural enemies, which should, in turn, exert higher mortality pressure on their prey (Root, 1973). A high level of heterogeneity is thought to provide more and alternative food resources, refuges, and shelter used for predators when hiding or nesting (Bereczki, Ódor, Csóka, Mag, & Báldi, 2014; Letourneau, Bothwell Allen, Kula, Sharkey, & Stireman, 2015). In addition, higher heterogeneity could favor the coexistence of multiple enemies (Amaral, Venzon, Perez, Schmidt, & Harwood, 2015; Finke & Denno, 2002), thus increasing top‐down mortality of herbivores.

Both for a basic understanding and for any sustainable application, it is important to have knowledge of the actual mechanisms linking predation pressure to habitat heterogeneity. The heterogeneity of a given habitat can be assessed by taking into account the parameters that characterize the vegetation and the dead plant material present, which in turn determine habitat complexity through structure and diversity (Stein & Kreft, 2014). Previous studies have focused mainly on the diversity of the plant species composing the vegetation (Björkman, Hambäck, Hopkins, & Rämert, 2010; Cappuccino, Lavertu, Bergeron, & Régnière, 1998; Riihimäki, Kaitaniemi, Koricheva, & Vehviläinen, 2005; Schuldt et al., 2011). However, other parameters linked to habitat heterogeneity, such as vegetation density (Warfe & Barmuta, 2004) or cover (Kotler, Brown, & Hasson, 1991) and understory vegetation height (Sobek, Tscharntke, Scherber, Schiele, & Steffan‐Dewenter, 2009), have been studied less. Therefore, more studies are required to consider multiple parameters contributing to habitat heterogeneity that may act synergistically to increase natural enemy pressure on herbivores.

Generalist and specialist predators are both important natural enemies of pest species, and their impact can reduce the likelihood of extreme fluctuations. Specialists, on one hand, play an important role when herbivore densities are already high, as their numerical response commonly leads to the prey—and the specialist predator—population crashing (Hassel & May, 1986). From a plant protection perspective, the effect of specialists may, however, come too late (Snyder & Ives, 2003) when plant damage is already severe. Generalist natural enemies, on the other hand, commonly have a more constant presence and a relatively higher abundance compared to specialists, as generalists are not dependent on one or a few prey species. A more stable abundance of generalist predators is thought to result in more stable predation pressure over time, which aids prevention of an increase in prey abundance and severe pest damage (Klemola, Tanhuanpaa, Korpimäki, & Ruohomaki, 2002; Pekár, Michalko, Loverre, Líznarová, & Cernecká, 2015).

As habitat heterogeneity is thought to be associated with high diversity (Stein, Gerstner, & Kreft, 2014) and density of alternative prey items (Stephan et al. 2015), this should benefit generalist predator abundance and thus pest population suppression. Generalist predators should be more efficient in maintaining prey populations at low densities as they are opportunistic and switch to the most easily obtained (often connected to highest abundance) prey at any given time (Murdoch, 1969). Thus, by switching, the generalists increase the mortality of an increasing population of prey and slow population growth, which in the long run leads to a reduction in magnitude of population fluctuations over time.

Small mammals are generalist predators thought to play an important role in forest ecosystems by regulating the population dynamics of a variety of herbivorous insects (Hanski, 1987; Tanhuanpää, Ruohomäki, Kaitaniemi, & Klemola, 1999). Variation in insect abundance may affect the regulating role of small mammals as these predators show a strong positive density‐dependent functional response up to a certain threshold prey density at which the response switches to become negatively density‐dependent (Type III; Holling, 1965). In addition, the abundance of small mammals is likely to be affected by habitat heterogeneity, for example, complexity (Carey & Harrington, 2001), tree species composition (Coppeto, Kelt, Vuren, & Van Wilson, 2006; Liebhold, Higashiura, & Unno, 1998), and shrub cover (Arnan, Comas, Gracia, & Retana, 2014). Dead wood is also considered an important component of habitat heterogeneity and found to be a key element for the conservation of a variety of living species (Lonsdale, Pautasso, & Holdenrieder, 2008), including many red‐listed species (Jonsell, Weslien, & Ehnström, 1998). The presence of dead wood is also to be important for small mammal presence (Manning & Edge, 2008). Increased habitat heterogeneity, including dead wood, offer protection to small mammals when consuming food, thus facilitating their feeding activity (Kollberg, Bylund, Huitu, & Björkman, 2014). Further, tree species composition and understory vegetation could provide alternative food resources such as seeds and fruits.

**Figure 1:**
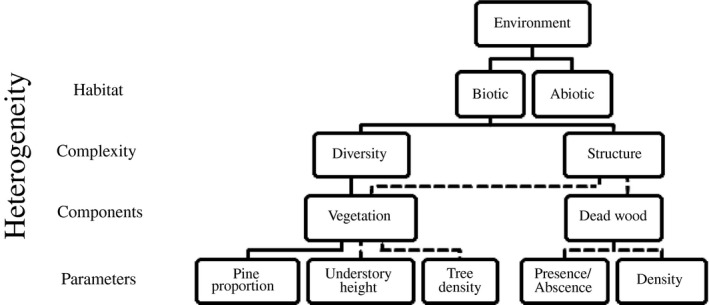
Representation of the levels of heterogeneity considered in this study. Solid and dotted lines represent parameters and components contributing to diversity and structure, respectively. The figure is based on Stein and Kreft (2014)

We here investigate the relationship between forest heterogeneity and small mammal predation on the European pine sawfly (*Neodiprion sertifer*, Geoffroy) cocoons. This species exhibits irregular outbreak dynamics in seminatural pine forests, and its outbreaks can cover thousands of hectares and last for several years (Lyytikäinen‐Saarenmaa & Tomppo, 2002). Small mammals, as predators of sawfly cocoons, contribute significantly to the population dynamics of this species (Hanski & Parviainen, 1985; Olofsson, 1987), and previous studies have found a positive relationship between habitat type and the abundance of small mammals (Ecke, Lofgren, & Sorlin, 2002; Sheftel & Hanski, 2002). In the study presented here, we investigated the effect of habitat heterogeneity on cocoon predation by small mammals over a period of two summers in a predator–prey system in which both the predator and prey can exhibit large fluctuations. First, we measured predation rates within forest stands that differed with respect to aspects of heterogeneity (Figure 1) such as pine proportion, tree density, and the amount of dead wood. We hypothesized that predation on European pine sawfly cocoons is higher in forest stands with high tree diversity. Second, we used an experimental approach to quantify how dead wood abundance affects small mammal predation and to test the hypothesis that a high density of dead wood will increase cocoon predation rate through increased small mammal activity.

## 2 MATERIALS AND METHODS

### 2.1 Study area

The study was carried out in forest stands between Björklinge (60°1′54.35″N, 17°33′7.58″E) and Månkarbo (60°13′36.08″N, 17°27′52.40″E), in the Uppland region in Sweden. The area is characterized by seminatural forests dominated by Scots pine (*Pinu*s *sylvestris* L.). Norway spruce (*Picea abies*) and silver birch (*Betula pubescens*) occur over the whole area, while rowan (*Sorbus aucuparia*), juniper (*Juniperus communis*), and oak (*Quercus robur*) are more rare with a scattered distribution.

### 2.2 Classification of the stands and selection of experimental pines

In the study area, we identified 10 different stands representing different levels of vegetation heterogeneity based on tree species diversity. Homogenous stands were dominated by Scots pine and were also characterized by the presence of mosses, lichens, heather (*Calluna vulgaris*), blueberry (*Vaccinium myrtillus*), and lingonberry (*Vaccinium vitis‐idaea*). Heterogeneous stands, in contrast, were characterized by different tree species and dominated by grasses (Poaceae) and ferns (e.g., *Pteridium aquilinum*).

For the selection of the experimental trees within the stands, a grid of 150 × 90 m was laid out and divided into 15 cells (30 × 30 m) in each stand. Within each grid cell, the pine tree closest to the center was selected to be the experimental tree. Around each experimental pine (area of 4 m radius ≈ 50 m^2^), we collected data on habitat heterogeneity measured as the number and species of trees, presence or absence of dead wood, and understory vegetation species (Table 1). For the latter, we determined the mean height and the coefficient of variation of the understory vegetation height based on four random height measurements around each experimental tree. These parameters were also used to obtain a description of the stands in which we performed our study (Table 1). The pine proportion (the number of pine present divided by the total number of trees) surrounding each experimental pine was used to classify the stands, as either monoculture (pine proportion >80%) or mixed forest stands (pine proportion <80%) following the method of Toumey and Korstian (1947). The stands were categorized based on the presence or absence of dead wood. If dead wood was present around half or more of the experimental pines, the stand was classified as “high dead wood density,” and those stands with fewer pines that had associated dead wood were classified as “low dead wood density.” Current management in Swedish forests does not specify the amount of dead wood required to leave within stands (FSC Sweden, 2010). Logs, high stumps, and wood debris are often left for conservation purposes but the amount is determined by the individual forest owner. Overall, tree density in a stand was calculated using the mean of the number of trees surrounding experimental pines.

**Table 1:** Site Description

Site	Coordinates	Experiment*	Stand classification	Plant density**	Pine proportion** (%)	Dead wood amount	Understory type***	Stand age**
1	60°6′33.71″ N 17°32′17.38″E	1,2	Mixed	33.8 ± 9.7	31 ± 15	Low	G,F	7.4 ± 0.5
2	60° 6′40.67″N 17°31′48.75″E	1	Mixed	22.4 ± 5.6	60 ± 18	Low	G,F	9.6 ± 1.1
16	60°10′40.36″N 17°29′22.44″E	1	Mixed	18.3 ± 11	67 ± 29	Low	G, F	7.7 ± 1.4
9	60° 8′58.09″N 17°29′34.52″E	1,2	Mixed	25.6 ± 9.4	50 ± 17	High	G,F	8.2 ± 1.2
11	60° 9′36.21″N 17°29′29.31″E	1,2	Mixed	34.3 ± 9.07	60 ± 19	High	G, F, M	6.8 ± 0.4
13	60° 9′58.20″N 17°29′16.06″E	1,2	Mixed	34.6 ± 9.6	69 ± 9	High	G, F, M	7.8 ± 0.8
3	60° 6′47.91″N 17°31′34.93″E	1	Mono	25.7 ± 4.5	90 ± 6	High	M, L, C, Li	10.2 ± 0.6
5	60° 7′13.71″N 17°30′43.88″E	1,2	Mono	23.3 ± 6.9	97 ± 3	High	M,L,C	7.6 ± 1.2
15	60°10′32.81″N 17°29′15.05″E	1,2	Mono	25.9 ± 9.9	91 ± 14	Low	M, L, C	11 ± 1.4
17	60°10′23.22″N 17°29′52.18″E	1,2	Mono	12.8 ± 6.7	96 ± 6	Low	M, L, C, B, Li	11 ± 1.2

*Experiment 1 = Habitat heterogeneity experiment; 2 = Dead wood manipulation experiment; **Sites 1 =  mean ± SD for 14 experimental pines; sites 2/3/16 = mean ± SD for 10 experimental pines; Sites 5‐9‐11‐13‐15‐17 = mean ± SD for 15 experimental pines; ***Grass = (G); Ferns (F); Moss (M); lichen (L); Calluna (C); Blueberry (B); Lingonberry (Li)

### 2.3 Neodiprion sertifer

The European pine sawfly, *Neodiprion sertifer* (Geoffroy, Hymenoptera, Diprinonidae), is a specialist herbivore on pine. The female lays her eggs in one batch during late August–early September, on needles of current year shoots, where the eggs overwinter until hatching in May. After hatching, the larvae feed gregariously on the needles from the previous year shoots until the fourth (male) or fifth (female) instar. At the end of June, the last instar larvae molt into prepupae, which leave the tree to spin cocoons in the forest topsoil. Predation by small mammals during the cocoon phase is thought to play an important role in the regulation of the pine sawfly densities (Hanski & Parviainen, 1985; Olofsson, 1987). In our study area, we assumed that the most common species of small mammals were the common shrew (*Sorex araneus*) and the bank vole (*Myodes glareolus*). Within the stands, sawfly larvae occurred at very low density. Cocoons for the experiments were collected by caging larval groups, and no naturally occurring cocoons were found during the experiment.

### 2.4 Habitat heterogeneity experiment

We used an experimental approach to test the effect of habitat heterogeneity on cocoon predation within the stands. The experiment was performed in summer 2014 at the end of August. Using the aforementioned grid, we randomly selected five of the potential fifteen trees in each experimental stand for a total of 50 pines. Around these pines, we placed three sets of cocoons 1–2 m away from each other: one single cocoon, one group with 10 and one group with 50 cocoons, totaling 3050 cocoons. The reason for using three different group sizes was to investigate how this affected predation and predator behavior. The cocoons were placed below the most superficial organic soil layer or under the understory vegetation; all remaining cocoons were collected after two weeks. We inspected the remaining cocoons for signs of predation, that is, teeth marks from small mammals or damage by arthropod predators, these and the missing cocoons were considered preyed upon by predators.

### 2.5 Dead wood manipulation experiment

To assess the effect of dead wood on cocoon predation rate, we performed a manipulation experiment at the end of July 2015. Again, using the aforementioned grid, we randomly selected ten of the potential fifteen trees in seven experimental stands. Within each stand, we recorded the amount of dead wood present around the selected trees dividing them into five groups of two trees with similar amounts of dead wood. Around one of the two trees in each group, we removed the dead wood, around the other tree we added dead wood, subjecting all experimental areas to disturbance. To quantify the amount of wood to add, we identified the biggest pile of dead wood in each stand, we weighed the pile (digital scale; Fladen, MH1625, 40 kg), and the volume was estimated using 70‐L bags. Subsequently, we collected the same amount of dead wood to add to the tree around where the wood was not removed. Consequently, the amount of dead wood around these trees exceeded the largest pile found and measured previously within the stand. The main component of dead wood was branches from previous cutting. The wood was divided into two piles on opposite sides of each tree that was selected for the “addition” treatment. The area of the dead wood around each of the “addition” trees was quantified based on pictures taken from above using ImageJ^®^ (Schneider, Rasband, & Eliceiri, 2012). The volume was obtained by multiplying the area by the mean of four randomly measured heights of the debris. Two groups of 15 cocoons were then placed between each experimental tree and the wood piles in the same manner as described in the previous experiment. The corners of a 0.86 × 0.86 m frame were used to place the cocoons in four smaller groups (4 + 4+4 + 3 cocoons per frame, in total 2100 cocoons). The edge of the frame was marked inconspicuously with a stone to enable the retrieval of the remaining cocoons. We collected the remaining cocoons after two weeks and the number of missing cocoons plus cocoons showing signs of predation (see description previous experiment).

### 2.6 Data analyses

#### 2.6.1 Habitat heterogeneity experiment

We performed three separate analyses to test (1) the difference in detection of cocoon groups of different size, (2) the probability that all cocoons within a group were entirely preyed upon, and (3) the predation rate, based on the proportion of cocoons preyed upon in relation to forest stand heterogeneity.

In the first analysis, the response variable included was the number of groups detected or not in a log‐linear model for contingency tables for count data using Poisson distribution (Crawley, 2007 pp 598–627). The categorical explanatory variables included were the size of cocoon groups, that is, 1, 10, or 50, the level of detection (yes or no) and their interaction. We considered a group detected when at least one cocoon was preyed upon. This analysis did not allow inclusion of heterogeneity parameters.

In the second analysis, we used the total (more than 80%) or no predation as a response variable in a logistic regression using a generalized linear mixed model with penalized quasi‐likelihood estimation (glmmPQL; Package MASS, Venables & Ripley, 2002) to account for data overdispersion using binomial error distribution. We considered a predation level within group of 80% or more (in groups of 10 and 50 cocoons) as “entirely preyed upon” and lower than 80% (in groups of 10 and 50 cocoons) as “no predation”. This percentage was set following the method described by Hanski and Parviainen (1985) based on the frequency distribution of cocoons preyed upon within groups that is similar to our finding (Fig. S2).

The third analysis included the response variable proportion of cocoon predation (continuous variable) within each group in a generalized linear mixed model with penalized quasi‐likelihood estimation (glmmPQL; Package MASS, Venables & Ripley, 2002) to account for data overdispersion using binomial error distribution. For the second and third analysis, the explanatory variables were the parameters related to forest structure around each experimental pine. Pine proportion and the natural logarithm of tree density were included as continuous explanatory variables. Group size and the presence/absence of dead wood around each tree were included as categorical explanatory variables. The experimental pine identity nested in site identity was included as random grouping factor. In both analyses, we included interactions between pine proportion and the other explanatory variables. To obtain the minimum adequate model, we used model simplification by backward elimination of nonsignificant variables starting with the interactions (Crawley, 2007;  pp. 388–448) using the type II test ANOVA (Package car, Fox & Weisberg, 2011). The Type II test ANOVA was selected to avoid that the order of explanatory variables in the model affects the final result. Type II ANOVA conforms to the marginality principle, assuming that the main effect of explanatory variables is marginal when these variables interact. In addition, we performed a Tukey post hoc test to reveal statistical differences between the different categories of group size.

#### 2.6.2 Dead wood manipulation experiment

We measured cocoon predation and variables quantifying diversity around each experimental pine. To analyze the effect of the amount of dead wood on the predation of sawfly cocoons, we used a generalized linear model with quasi‐binomial error distribution to account for data overdispersion. The two groups of 15 cocoons around each experimental pine were merged and used as the response variable. Site identity was included as a blocking factor while wood treatment was included in the model as a categorical explanatory variable. The continuous explanatory variables included were the natural logarithm of dead wood volume, the proportion of pines, the tree density surrounding each experimental pine, and the coefficient of variation calculated for understory vegetation height. As we were interested in the role of dead wood, we included only the interactions between dead wood treatments and the explanatory variables mentioned to test possible effects on cocoon predation. To obtain the minimum adequate model, we used the same procedure as for the previous experiment.

## 3 RESULTS

### 3.1 Habitat heterogeneity experiment

The first analysis showed that cocoon groups of different size differed in their detectability (χ^2^ = 45.4, *df* = 2, *p*‐value < .001). The groups with 50 cocoons were always detected while groups with 10 cocoons had a probability of 76% to be detected and single cocoons only 48% to be found and preyed upon (Table 2). The second analysis revealed that when detected, a group with 50 cocoons had 24% chance to be entirely preyed upon. This probability increased with the decrease in group size, the group of 10 cocoons had a probability of 37% to be entirely preyed upon (Tables 2, 3). However, only the difference between the group of 50 cocoons and the single cocoon was significant (*p* = .03). The second analysis revealed that the probability of being entirely preyed upon increased with increasing tree density, that is, number of trees surrounding experimental pines (Figure 3, Table 3). In the presence of dead wood, the probability for a group to be entirely preyed upon significantly decreased with increasing proportion of pine trees, whereas in the absence of dead wood, the probability increased with increasing pine proportion (Figure 4, Table 3). The results of the third analysis showed that cocoon groups of different sizes do not differ in the amount of predation. Instead, the number of cocoons fed upon is positively related to density of trees (log_e_) surrounding the experimental pines (Figure 5, Table 4).

**Table 2:** The table shows, for each cocoon group size, the count of pine and the probabilities for detectability (at least one cocoon preyed upon) and if they were entirely preyed upon (>80% for the group of 10 and 50)

Group size	Detectability		Entirely preyed upon	
	Count	Probability (%)	Count	Probability (%)
1	24	48	24	48
10	38	76	22	37
50	50	100	19	24

Total number of pine for each group size was 50

**Table 3:** ANOVA (type II test) and summary table for generalized linear mixed effect model with penalized quasi‐likelihood testing the probability of predation on sawfly cocoons in relation to forest diversity within stands

Fixed effect	Est	SE	χ^2^	*df*	*p*‐value
Intercept	−6.39	2.81			
Groups			7.08	2	**.02**
1	−0.05	0.63			
10	−0.49	0.42			
50	−1.14	0.43			
Tree density (log_e_)	1.87	0.78	5.9	1	**.01**
Pine prop			0.11	1	.72
Dead wood			0.29	1	.58
Pine prop: Dead wood			4.2	1	**.04**
Absence	3.7	0.9			
Presence	−3.8	0.5			
Random effect	Intercept	Residuals			
Site identity	0.93				
Site identity/Pine identity	2.63	0.61			

Table shows the final model with the estimates, the standard error of the mean (SE), chi‐squared value, degrees of freedom (*df*), and the *p*‐value for fixed predictors and the intercept and the residuals of the random effects. Estimates and standard error are shown only for significant predictor and interaction. Values are obtained using sum contrast as there was no natural way to set a baseline or an ordering in the different levels of the variables. Total number of observations: 150

**Table 4:** ANOVA (type II test) and summary table for generalized linear mixed effect model with penalized quasi‐likelihood testing the proportion of preyed upon sawfly cocoons in relation to forest diversity within stands

Fixed effect	Est	SE	χ^2^	*df*	*p*‐value
Intercept	−2.75	1.68			
Group			1.75	2	.41
Tree density (log_e_)	1.24	0.50	5.2	1	**.02**
Pine prop			0.16	1	.68
Dead wood			0.13	1	.7
Pine prop: Dead wood			3.00	1	**.08**
Absence	2.92	3.5			
Presence	−2.29	2.5			
Random effect	Intercept	Residuals			
Site identity	0.9				
Site identity/Pine identity	2.25	1.89			

Table shows the final model with the estimates, the standard error of the mean (SE), chi‐squared value, degrees of freedom (*df*), and the *p*‐value for significant predictors and the intercept and the residuals of the random effects. Estimates and standard error are shown only for significant predictor and interaction Values are obtained using sum contrast as there was no natural way to set a baseline or an ordering in the different levels of the variables. Total number of observations: 150

### 3.2 Dead wood manipulation experiment

There was no direct effect of dead wood addition or removal on predation rates (Table 5). But we did find a significant interaction between the proportion of pine and the dead wood treatment, showing that an increase in the proportion of pine at a site had a negative effect on predation rate when dead wood was removed, indicating that predation rates declined more when dead wood was removed in an environment that otherwise would not provide much shelter opportunities. Adding dead wood decreased predation rates when the proportion pine increased, but this relationship was less steep (Figure 6). None of the other main effects or interactions were significant.

**Table 5:** ANOVA (type II test) and summary table for linear mixed effect model testing the importance of dead wood for predation of sawfly cocoons by small mammals. “Treatments” represent the two treatments around experimental pines (wood addition, wood removal)

Predictor	Est	SE	χ^2^	*df*	*p*‐value
Intercept	1.49	1.13			
Site			24.01	6	**<.001**
Tree density			0.18	1	.6
Dead wood volume (log_e_)			0.6	1	.41
CV understory veg. height			0.05	1	1.08
Treatments			1.3	1	.25
Pine prop			1.4	1	.23
Treatments: Pine prop.			6.1	1	**.012**
Wood addition: Pine prop	−0.7	0.64			
Wood removal: Pine prop	−3.54	0.90			

Table shows the final model with the estimates, the standard error of the mean (SE), chi‐squared value, degrees of freedom (*df*), and the *p*‐value for significant predictors. Values are obtained using sum contrast as there was no natural way to set a baseline or an ordering in the different levels of the variables. Total number of observations: 63

## 4 DISCUSSION

Our main goal was to elucidate the link between cocoon predation and habitat heterogeneity. Contrary to our expectation, tree species diversity did not affect cocoon predation, but tree density increased the probability of cocoons being entirely preyed upon as well as the proportion of cocoons preyed upon. Our second hypothesis, testing the benefits of dead wood on cocoon predation, was partially supported as the effect of dead wood presence on cocoon predation varied with tree diversity in the stand. Our study showed that habitat heterogeneity resulted in increased mortality of herbivores through higher predation pressure.

**Figure 2:**
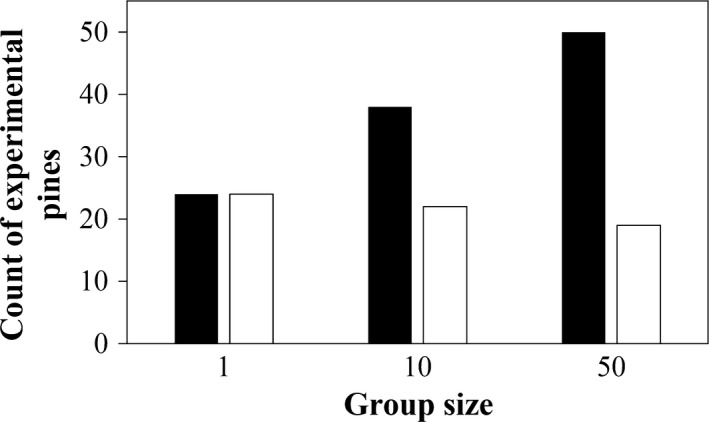
Count of experimental pines where sawfly cocoon groups have been detected (black bars; at least one cocoon preyed upon) and entirely preyed upon (white bars; >80% for the group of 10 and 50). For each group size, the total number of experimental pines was 50

In our first experiment, a single cocoon showed a 48% chance of being detected and preyed upon compared to a 24% chance if instead the cocoon was part of a larger group (Figure 2). The higher survival probability occurring within a large group is part of a mechanism known as the “dilution effect” (Turner & Pitcher, 1985) and as shown for *Lymantria dispar* cocoons (Gould, Elkinton, & Wallner, 1990). However, under natural conditions, sawfly larvae spin their cocoon solitarily, which implies that other factors, additional to predation risk, have driven the evolution of this life history trait. For instance, during the short time period (2 weeks) studied here, the encounter rates of a cocoon within a group might be different compared to a longer time period (6–8 weeks) in which sawfly cocoons are normally exposed. A longer exposition period could increase the encounter rate of cocoons with natural enemies annulling the dilution effect. In addition, variation in natural enemy density probably affects the chance of being preyed upon both for single cocoons and cocoons within a group. Another factor that is likely to affect the chance of a cocoon to be preyed upon is habitat heterogeneity; high heterogeneity could be expected to indirectly promote the predation of cocoons by increasing the presence of small mammals through higher abundance of food resources related to vegetation (seeds, berries) or dead wood (fungi, lichens, insects) (Ecke et al., 2001). To what extent the likelihood of single cocoons and a cocoon in a group to be preyed upon is affected by habitat heterogeneity needs further study. In addition, abundant alternative food may at the same time reduce the probability that single or group of cocoons will be completely preyed upon.

**Figure 3:**
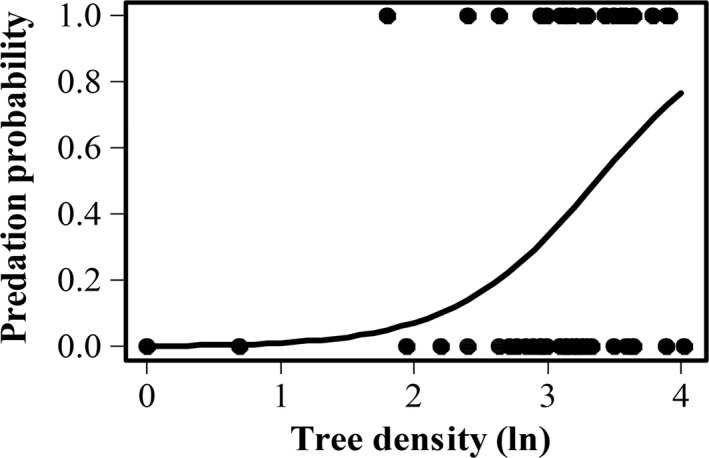
Probability of sawfly cocoons being preyed upon in relation to the natural logarithm of tree density surrounding the experimental pines. Solid curve is fitted generalized liner mixed model with binomial error distribution. The number of observations was 150 (50 experimental pines, three categories group size), points might overlap

**Figure 4:**
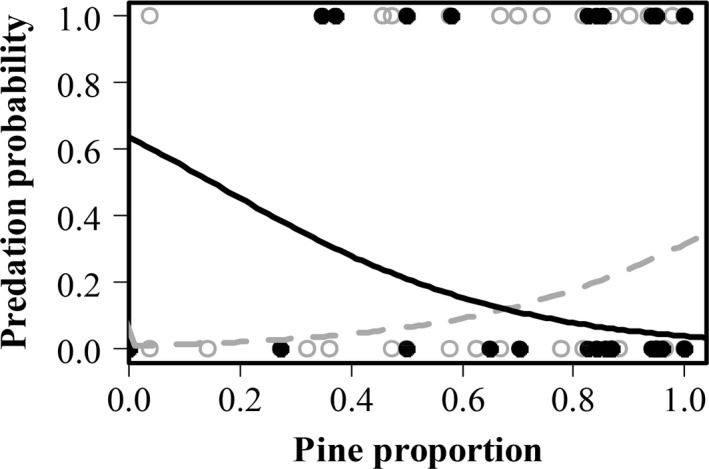
Probability of sawfly cocoons being preyed upon in relation to two categories of dead wood amount and proportion of pines. Solid line and filled points represent presence of wood, dotted line and empty points represent absence of dead wood. Solid and dotted curves are fitted generalized liner mixed model with binomial error distribution. The sample size was 150 (50 experimental pines, three categories of group size), points might overlap

**Figure 5:**
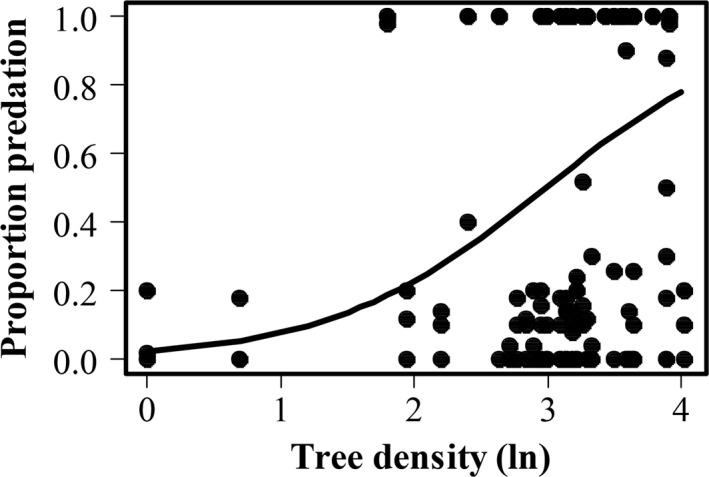
Proportion of sawfly cocoons preyed upon in relation to the natural logarithm of tree density surrounding the experimental pines. Solid curve is fitted generalized liner mixed model with penalized quasi‐likelihood estimation. The number of observations was 150 (50 experimental pines, three categories of group size), points might overlap

The mechanisms controlling the ways in which variation in habitat heterogeneity could affect cocoon predation include effects on the behavior and density of small mammals. For instance, it has been shown that small mammals exhibit a preference for habitats that offer shelter from their enemies (Ecke et al., 2002; Sundell, Church, & Ovaskainen, 2012); this includes stands with high tree density. Furthermore, our result shows that patches with high tree density positively affect cocoons predation rates by small mammals, thus cocoons in these dense areas within a stand are at higher risk of predation (Figure 3, 4, 5).

**Figure 6:**
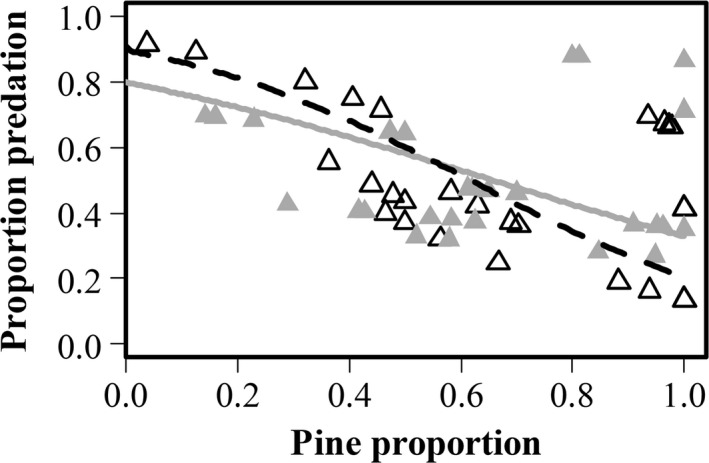
Proportion of sawfly cocoons preyed upon in relation to two dead wood manipulation treatments and the proportion of pines around each experimental pine. Solid line and filled gray points represent the wood addition treatment while dotted line and empty points represent wood removal treatment. The number of observations was 63, points might overlap

Dead wood contributes to habitat heterogeneity (McElhinny, Gibbons, Brack, & Bauhus, 2005) providing protection for small mammals when feeding and thus have the potential to increase their feeding activity (Kollberg et al., 2014). Our results show that there is an overall negative effect on the cocoon predation rate of the proportion of pine around the experimental tree, but the negative relation became less steep when dead wood was added (Figure 6). This difference in decrease can be caused by the effect of small mammal behavior as well as variations in natural conditions of forest stands that differ in pine proportion. The higher predation rates in stands with a higher tree diversity (i.e., lower proportion of pine) might be the result of rich habitats that provide more alternative food sources allowing high densities of small mammals (Sullivan & Sullivan, 2012). In contrast, in stands with a high proportion of pine, which are often considered poor and dry habitats (Kouki, Lyytikäinen‐Saarenmaa, Henttonen, & Niemelä, 1998), wood piles may be important for small mammals because of the presence of alternative food associated with wood (Ecke et al., 2001). Dead wood removal in these poor stands could therefore lead to a reduction in small mammal presence and consequently a decrease in cocoon predation.

The potential positive effects of forest heterogeneity as considered in our experiments on cocoon predation should be interpreted with caution. Firstly, a strong correlation between, for example, tree diversity and understory vegetation makes it difficult to evaluate the effect of the individual stand characteristics. Another confounding factor is that soil type will set the limits for the kind of tree species that potentially can occur at a specific site, in turn, reducing the number of possible forest management options. However, these confounding factors represent the natural variation in forest condition available for our experiment. Because these factors are difficult to control for, we focused on a parameter that is easily manipulated (i.e., dead wood), which made our results easier to interpret and also better to apply in management strategies. Manipulating dead wood is a management measure that is also connected to species conservation, making active management of dead wood in forest stands a win–win solution.

Thus, our study suggests that appropriate management of components contributing to heterogeneity in forest stands could positively affect the pressure exerted by small mammals on pest insects. But confounding factors, such as stand diversity and understory vegetation, need to be considered as the separate effects of these factors are difficult to tease apart. The conclusion that management decisions may actively affect the potential for control of insect populations, for example, dead wood retention, still remains valid. For forest management, benefits of dead wood retention are therefore two‐sided, potential pest regulation, and conservation benefits, but the effect may vary over time due to fluctuations in prey and predator abundance. Thus, our study is one of few that empirically have studied how forest management might affect top‐down control of insect pests. However, in order to maximize the effects of any management decisions, it would be valuable to identify the habitat heterogeneity factors that have the largest positive effects on generalist predators, thus reducing the risk of insect outbreaks.

## CONFLICT OF INTEREST

None declared.

## AUTHOR CONTRIBUTIONS

All authors developed the ideas and the study design. DB executed the experiment and DB and MJK analyzed the data, and the figures were designed by DB. DB led the writing of the manuscript to which all authors contributed and gave their consent for publication.
