# Supplementary figures and images for: Habitat heterogeneity affects predation of European pine sawfly cocoons
Source: Ecol Evol. 2017 Nov 12;7(24):11011–20. doi: 10.1002/ece3.3632 (PMC5743652; doi:10.1002/ece3.3632)

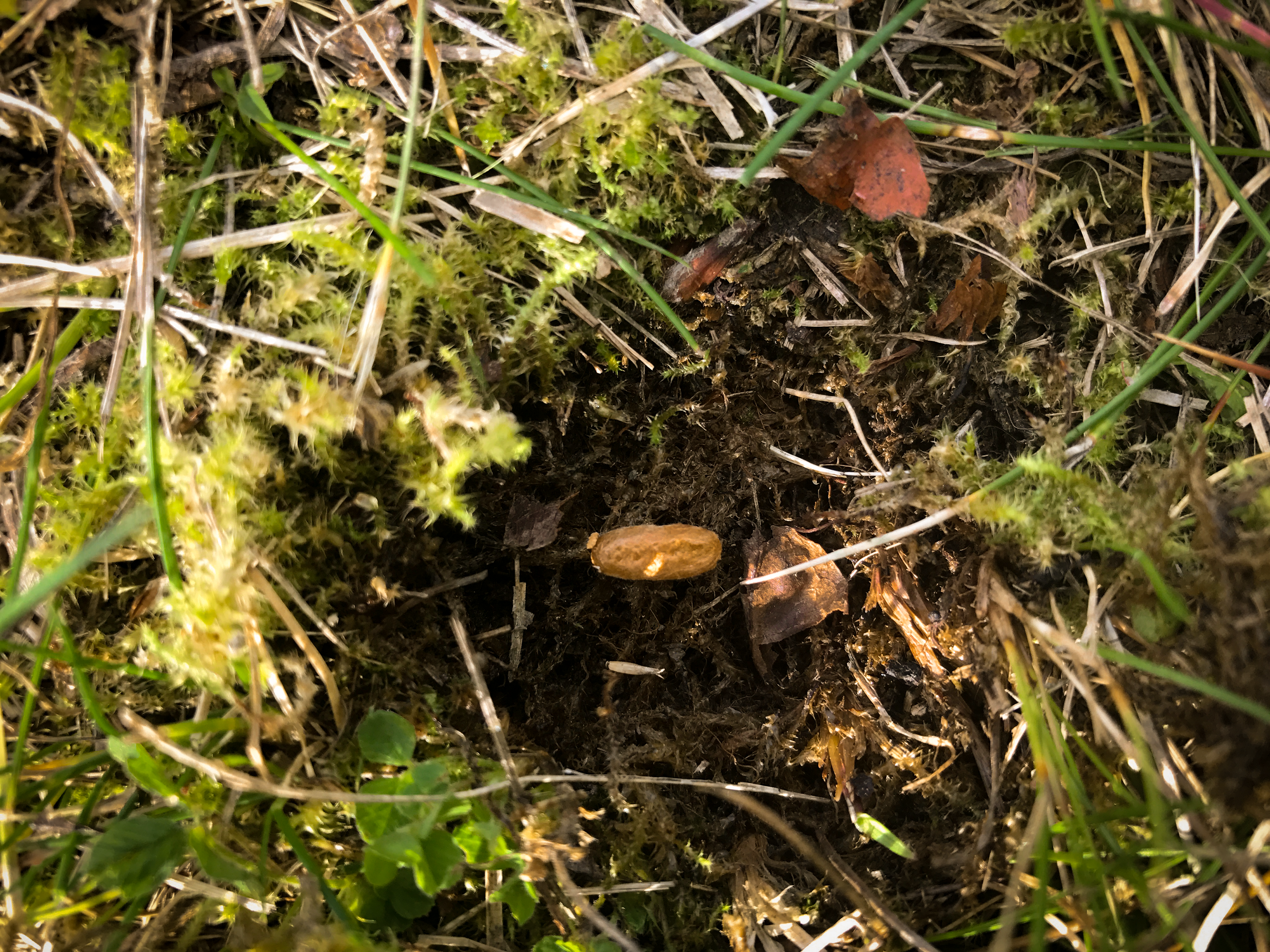

Supplement: Supplementary file 1 [file ECE3-7-11011-s001.png]
